# Antibiotic hypersensitivity and adverse reactions: management and implications in clinical practice

**DOI:** 10.1186/s13223-020-0402-x

**Published:** 2020-01-21

**Authors:** Anthony Jourdan, Brijit Sangha, Eric Kim, Sohrab Nawaz, Vikram Malik, Radhika Vij, Sudhir Sekhsaria

**Affiliations:** 1 Georgetown University School of Medicine, Washington DC, USA; 2 Medstar Union Memorial Hospital, 3333 N Calvert St, Suite 520, Baltimore, MD 21218 USA

**Keywords:** Antibiotic, Hypersensitivity, Adverse reactions, Allergy, Cutaneous reactions, Penicillin, Anaphylaxis

## Abstract

**Background:**

Studies have shown the discrepancy between self-reported antibiotic allergies and true allergies. Inaccurate reporting of antibiotic hypersensitivities can limit treatment options and result in use of more expensive antibiotics and contribute to resistance.

**Methods:**

This retrospective cohort chart review obtained data on 16,515 patients after obtaining IRB approval. Patients who had an antibiotic adverse reaction were identified, recorded, and their management reviewed. 7926 patients were selected from inpatient internal medicine clinics, 8042 patients from outpatient internal medicine clinics, and 547 from orthopedic clinics.

**Results:**

The prevalence of reported antibiotic sensitivity in our study was 9.89% (n = 1624). Reported antibiotic sensitivity was 8.88% (n = 704) in inpatient settings as compared to 11.2% (n = 902) and 5.12% (n = 28) in medicine and orthopedic outpatient settings respectively. The top five antibiotic adverse reactions reported were penicillins (42%), sulfonamides (25%), fluoroquinolones (4.3%), tetracyclines (4.2%), and macrolides (3.5%). In all settings, penicillins and sulfonamides adverse reactions were the top two reportings. 11.88% (n = 193) of patients with reported adverse reactions reported sensitivities to multiple antibiotics.

**Conclusion:**

Our study demonstrated high prevalence of reported antibiotic sensitivity in three clinical settings. However, a significant portion of these patients may not be truly hypersensitive to these antibiotics. There is a need for increased awareness among medical professionals about the importance of detailed history taking and management of self-reported antibiotic allergies to combat unnecessary use of antibiotics.

## Background

Antibiotics are among most commonly prescribed drugs given to patients to treat bacterial infections and mitigate bacterial growth. Though readily effective against bacterial pathogens, antibiotics can cause adverse drug reactions due to hypersensitivities in patients [1]. Though a patient can have an adverse reaction after administration of the antibiotic, an adverse reaction or hypersensitivity does not confer a true allergy to the medication [2]. Many patients self-report their symptoms to their physician for many of the known and unknown antibiotic sensitivities. In many instances these claims are unproven because adverse drug reactions can manifest in many forms, as there is a lack patient knowledge and there are time constraints in healthcare settings.

In 2015, the antibiotic prescription ratio to people was 838 prescriptions for every 1000 people in the United States [3]. With such high rates of antibiotic usage, the occurrence of adverse drug reactions or hypersensitivities from antibiotic usage becomes an important topic for healthcare professionals.

Antibiotic hypersensitivity can often be a result of the non-selective killing of the targeted bacteria. Some of the most common adverse reactions include symptoms such as diarrhea, nausea, vomiting, rashes, and gastrointestinal distress [2]. Such adverse drug reactions are immune system mediated, impacting various organ systems. The severity is affected by numerous factors such as drug characteristics including duration of use and strength, as well as environmental factors including the individual’s immune system [4]. These reactions are often classified into Type A and Type B reactions. Type A reactions are predictable in most cases and are usually caused by pharmacological adverse effects and drug interactions. Type B reactions are usually unpredictable and can either be immune mediated or non-immune mediated. Immune mediated reactions include IgG mediation, T cell mediation, and immune complex deposition. Though these are all immune mediated, true allergy is not indicated unless it is mediated via an IgE mechanism [5].

Antibiotic hypersensitivities are usually inadequately documented in official medical platforms, thus the majority of knowledge gained about these sensitivities is through the self-reporting from the antibiotic users [6]. In many cases, improper documentation of antibiotic hypersensitivities prevents patients from being able to use first line antibiotic medications [7]. These first line drugs are often more effective, possess fewer side effects, are narrower in range, and are more cost efficient [8]. Therefore, it is of key clinical interest to clinicians to have accurate documentation of antibiotic reported adverse reactions, the reactions and temporal context associated with these adverse reactions, and whether these reactions confer true allergy.

Previous studies have not compared the reported antibiotic sensitivities in outpatient versus inpatient clinical settings. It is possible that the reported antibiotic allergy could vary in these two settings based on the detailed history taken by the healthcare professional. This study focuses primarily on self-reported and documented antibiotic adverse reactions within three clinical settings. These settings include inpatient internal medicine clinics, outpatient internal medicine clinics, and orthopedic clinics across Baltimore, Maryland, and its surrounding metropolitan area. This study aims to provide prevalence data in regard to antibiotic hypersensitivity and reaction, analyze discrepancies in self-reports and documentations of hypersensitivities and true allergies, as well as synthesize trends in data to make informed decisions and propose solutions for management and treatment.

## Methods

To conduct this study, IRB approval was sought and granted by the MedStar Health Research Institute Institutional Review Board. Retrospective chart reviews were then undertaken from three clinical settings. These included inpatient internal medicine clinics, outpatient internal medicine clinics, and outpatient orthopedic clinics. 7926 patients were gathered from inpatient medicine clinics. 8042 patients were gathered from outpatient medicine clinics and 547 patients were gathered from orthopedic surgery clinics. Data was primarily collected from patients at Union Memorial Hospital and other MedStar Medical facilities throughout the Baltimore metropolitan area.

**Fig. 1:**
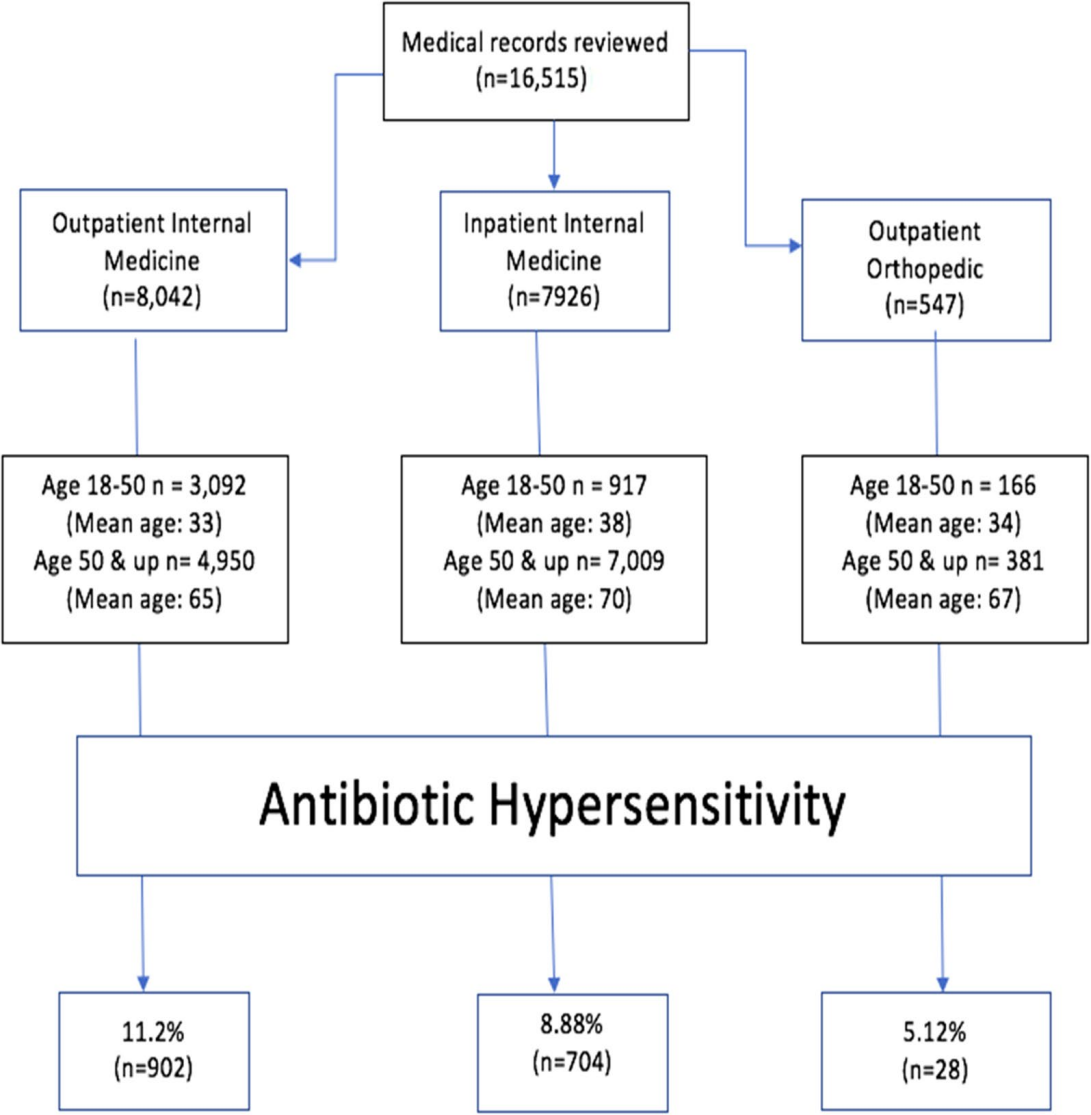
Flow chart of collection of data. Study design showing the number of patients with documented antibiotic hypersensitivity and the demographics of the population in regards to age. After collecting and reviewing medical records, the patients were separated by the type of care facility they received care. Date of birth information was collected to provide demographic insight of the cohort. Antibiotic hypersensitivity was then identified in these patients and prevalence data was synthesized

Patients with a documented antibiotic hypersensitivity were selected and their electronic medical records were thoroughly reviewed. The following information was collected: documentation of the reaction reported, what antibiotic was involved (Table 1**)**, and what type of reaction was reported or documented. The reactions were classified as either cutaneous, anaphylaxis, gastrointestinal manifestations and Not Reported. Figure 1 demonstrates the progression of the data collection with notable demographic information.

**Table 1:** Antibiotics of interest: allergies to these antibiotics denoted a patient to have an antibiotic hypersensitivity. Reporting of a hypersensitivity to any antibiotic was reported to an allergy of the associated class

Antibiotic class	Antibiotics within class
Penicillin	Amoxicillin, Amoxicillin–Clavulanate, Ampicillin, Ampicillin–Sulbactam, Nafcillin, Penicillin G Potassium, Penicillin VK, Piperacillin–Tazobactam, Dicloxacillin
Sulfonamides	Sulfamethoxazole–Timethoprim, sulfur
Fluoroquinolones	Levofloxacin, Ciprofloxacin
Cephalosporin	Cefaclor, Cefazolin, Cefepime, Cefixime, Cefoxitin, Ceftaroline, Cephalexin, Cefuroxime, Ceftriazone, Ceftazidime–Avibactam, Cefrolozane–Tazobactam
Macrolides	Azithromycin, Clartithromycin, Erythromycin
Tetracycline	Tetracycline, Doxycycline
Miscellaneous	Clindamycin, Vancomycin

Across all three settings, 4175 patients were under the age of 50 as compared to 12,340 patients over the age of 50. Within the inpatient internal medicine facilities, 917 patients were under the age of 50 with a mean age of 38. In these same facilities, 7009 patients were over the age of 50 with a mean age of 70. Within the outpatient internal medicine facilities, 3092 patients were under the age of 50 with a mean age of 33. In these same facilities, 4950 patients were over the age of 50 with a mean age of 65. In orthopedic settings, 166 patients were under the age of 50 with a mean age of 34. 381 patients were over the age of 50 in orthopedic settings with a mean age of 67.

## Results

The overall prevalence of adverse reactions in our cohort was 9.89% (n = 1624). In inpatient internal medicine practice, the prevalence of antibiotic hypersensitivity was 8.88% (n = 704). Compared to inpatient medicine setting, outpatient internal medicine practice was 11.2% (n = 902). Orthopedic Outpatient clinic settings reported a prevalence of 5.12%.

Across all three settings, the most commonly reported antibiotic involved was penicillin, reported by 42.11% (n = 684) of patients. The next most common antibiotic involved was to sulfonamides, reported by 24.8% (n = 402) of patients. Self reported adverse reactions for further antibiotic classes are as follows: fluoroquinolone (Ciprofloxacin and Levofloxacin) adverse reaction was reported by 4.3% (n = 70) of patients, tetracycline adverse reaction was reported by 4.2% (n = 69) of patients, macrolide adverse reaction was reported by 3.5% (n = 57) of patients, cephalosporin adverse reaction was reported by 2.3% (n = 38) of patients, vancomycin adverse reaction was reported by 1.9% (n = 31) of patients, and clindamycin adverse reaction was reported by 1.2% (n = 20) of patients. Finally, 11.88% of patients reported a adverse reaction to two or more antibiotics.

In inpatient internal medicine clinics, the most common antibiotic adverse reaction reported was to penicillins. This was reported by 31.1% (n = 219) of patients. Sulfonamide adverse reaction was the next most commonly reported by 14.4% (n = 102) of inpatient internal medicine patients. 4.6% (n = 33) of inpatient internal medicine patients reported an adverse reaction to fluoroquinolones (Ciprofloxacin and Levofloxacin), while 10.22% (n = 72) of patients reported a adverse reaction to two or more antibiotics.

In outpatient internal medicine clinics, penicillin and sulfonamide self-reported adverse reactions were again the most and second most commonly reported, at 35.9% (n = 324) and 17.4% (n = 157) respectively. The third most commonly reported adverse reaction was to macrolides at 3.2% (n = 29). 9.09% (n = 82) of patients in the outpatient medicine clinic reported adverse reactions to two or more antibiotics.

**Fig. 2:**
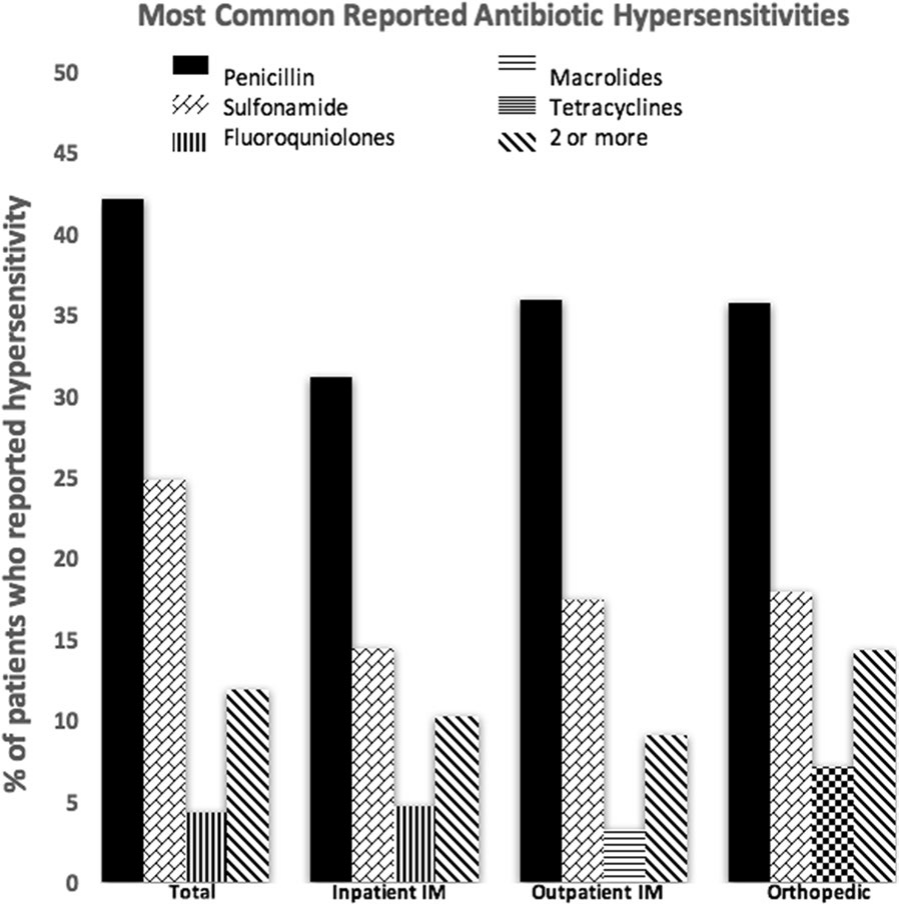
Most common reported antibiotic hypersensitivities. Hypersensitivities to specific antibiotics are noted above. The reportings are separated by all three settings (total), inpatient internal medicine, outpatient internal medicine, and outpatient orthopedic. The legend located within the inset of the graphs states which bars correspond to which antibiotic class

In orthopedic clinic settings, the three most common reported antibiotic adverse reactions were penicillins, sulfonamides, and tetracyclines, reported by 35.7% (n = 10), 17.9% (n = 5), and 7.1% (n = 2) respectively. 14.3% of patients in outpatient orthopedic clinics reported adverse reaction to 2 or more antibiotics. The data is represented graphically in Fig. 2.

**Fig. 3:**
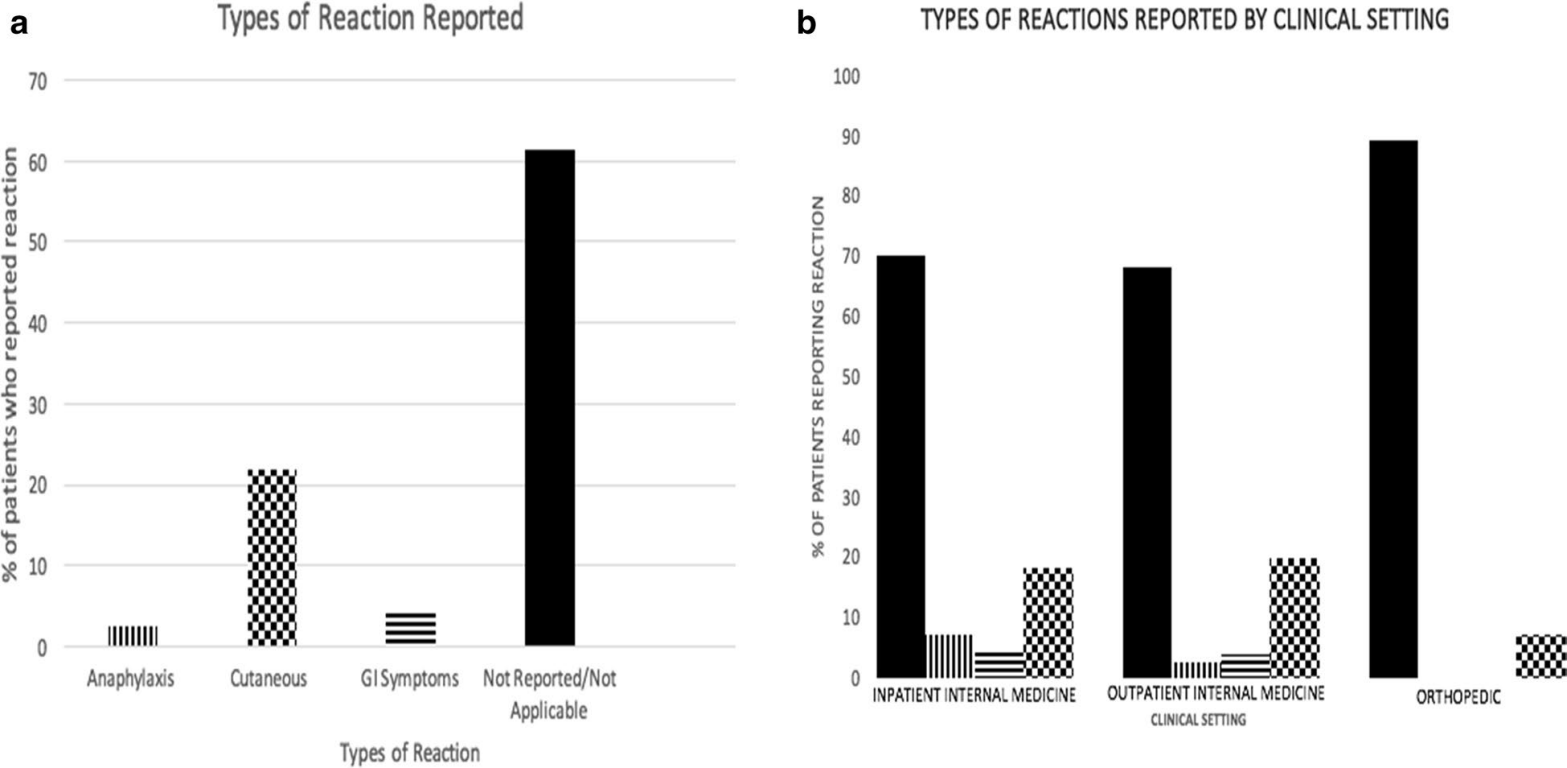
**a** Types of reaction reported in all three clinical settings. Reported reactions from patients across all three settings are reported. The reactions were classified into anaphylaxis, cutaneous reactions, GI symptoms, and Not Reported/Not Applicable. Cutaneous reactions include itching, redness, hives, angioedema, red man syndrome, rashes, and swelling. Not reported/Not applicable includes documentations of unknown and N/A. **b** Types or reaction reported by clinical setting. Reported reactions from patients at each of the three clinical setting categories. Bars are coordinated to the associated bar design in Fig. 3a. No reportings of GI symptoms or anaphylaxis occurred at outpatient orthopedic clinics

Across all three settings, the most commonly reported adverse reaction was cutaneous, reported by 21.8% (n = 355) of patients. Gastrointestinal symptoms were reported by 4.4% (n = 71) of patients, while anaphylaxis was reported by 2.5% (n = 41) of patients. 61.4% (n = 997) of patients records who reported an antibiotic adverse reaction were categorized as Non-Reported as specific reaction data was not documented (Fig. 3a).

In inpatient internal medicine clinics, cutaneous reactions accounted for 18% of all reactions reported, gastrointestinal symptoms accounted for 4.3%, and anaphylaxis accounted for 1.7%. 70.1% (n = 494) of patients records who reported an antibiotic adverse reaction were categorized as Non-Reported as specific reaction data was not documented. In outpatient internal medicine clinics, cutaneous reactions accounted for 19.7% of all reactions reported, gastrointestinal symptoms accounted for 3.9%, and anaphylaxis accounted for 2.5%. 68.1% of patients who reported a adverse reaction were categorized as Non-Reported as specific reaction data was not documented. In outpatient orthopedic clinics, cutaneous reactions accounted for 7.1% of reactions reported. 89.2% of patients who reported a adverse reaction were categorized as Non-Reported as specific reaction data was not documented. There was no reporting of gastrointestinal symptoms or anaphylaxis in outpatient clinics (Fig 3b).

## Discussion

Scientific literature has estimated that across all clinical settings, antibiotic adverse reaction prevalence is seen in 11% of patients [9]. This is similar to the determined prevalence in our study. However, our study demonstrates that there is no difference in the reported adverse reactions and hypersensitivities across three different clinical settings, inpatient medicine, outpatient orthopedic, and outpatient medicine. Additionally, penicillin hypersensitivity remains the most common hypersensitivity among antibiotics [10]. This was mirrored similarly in our study across all three settings and each respective clinical setting demonstrated that penicillin adverse reaction was the most common antibiotic adverse reaction reported. Systematic meta analyses have shown that outside the penicillins and beta lactams, sulfonamide drugs are the next most commonly reported antibiotic hypersensitivity reported by patients [11]. This similarly follows the data we obtained from our study cohort. The high rate of penicillin hypersensitivity reporting is of particular clinical interest. Penicillins are a cheap and effective antibiotic that are often used clinically to combat numerous infections ranging from common respiratory tract infections to more complicated infections like cellulitis [10]. Patients with a history of penicillin allergy are more likely to be treated with broad spectrum antibiotics. There are distinct disadvantages to these agents, which are often more expensive and associated with more serious side effects [10]. Therefore, the ability to identify true allergies is of interest to clinicians.

It has been suggested that 90% of all patients reporting a penicillin allergy can in fact take a penicillin class antibiotic [2, 12, 13]. This can be due to several reasons. First, patients can lose sensitivity to specific penicillin IgE antibodies over time if penicillin is properly avoided. Second, non-IgE-mediated immune reactions of skin or gastrointestinal tract are often wrongly attributed to an IgE-mediated process from a concurrent medication [14]. Therefore, to effectively determine if a patient is suffering from a true penicillin allergy, diagnostic tests should be used in conjunction with patient reported symptoms and observations from clinical professionals. Diagnostic tests that should be considered include but are not limited to measurement of allergic mediators (histamine, tryptase), allergen specific IgE levels, basophil activation assays, and cellular antigen stimulation test (CAST ELISA) [15].

Literature review shows that cutaneous skin reactions are the most commonly reported adverse drug reaction, followed by gastrointestinal manifestations. Anaphylactic reactions are reported less frequently than both [15]. This trend is noticeable in our cohort’s reporting, indicating similarity between our patient population and comparable populations in literature review. However, the notable aspect of our study was the large amount of non-reportings of adverse drug reactions across all three settings. This lack of reporting makes it more difficult for healthcare professionals to understand the underlying mechanism of the patient’s adverse reaction and identify whether a true allergy is present [16]. Mechanistically, if a reaction were to occur through an IgE mechanism, then we would expect this to happen rapidly. This would be identified as a true allergy. If the reaction were to take longer to manifest, then we would expect that it is a non IgE mediated drug allergy that is mediated by IgG or IgM, T cells, or drug immune complex reactions [15]. Finally, reportings of gastrointestinal distress are not signs of true allergy as well. Most manifestations are in fact dose related or adverse reactions and are not indicative of an IgE mediated process [17]. Simple changes in dosing can provide relief for these patients.

Incomplete patient histories and physical examinations can compromise patient care as many preventable medical errors are caused by poor documentation [18, 19]. For example, information such as reaction onset, duration, and characteristics of presentation are all vital in defining a true allergy and should be investigated by clinicians. To combat the challenges unearthed in our study, it is imperative that we make allergy questioning a necessary part of the patient encounter, and update as needed. Documentation should include what specific reaction occurred, along with the onset and length of the reaction, the number of doses taken by the patient, and the route of administration [20]. After consulting and learning more about the allergy, consideration for an allergist evaluation may be of benefit to the patient [21]. Careful documentation and investigation of a hypersensitivity is necessary as it can directly influence patient morbidity and mortality.

Our study has certain limitations. As a retrospective chart review, reporting and documentation at times were missing. We included patients with a history of antibiotic adverse reactions that was mostly self-reported or based on previous patient’s documentation, but not confirmed by dedicated diagnostic tests. The prevalence of true antibiotics hypersensitivity in this cohort might be overestimated.

## Conclusions

Antibiotics remain some of the most commonly prescribed drugs, and as such adverse reactions and hypersensitivity are inherent to their use. The cohort in this study displays characteristics similar to study populations evaluated during literature review. Penicillin adverse reaction remains the most commonly reported hypersensitivity, followed by sulfonamide adverse reaction. In addition, cutaneous manifestations were the most commonly seen adverse reactions. Delayed cutaneous reactions represent immune mediated hypersensitivities and gastrointestinal manifestations usually represent adverse reactions to antibiotics. However, these reactions often act as barriers to first line antibiotic use on the basis of being misidentified as true allergies. Due to various factors such as time constraints and lack of understanding, clinicians often miss opportunities to extract more information from patients about their reported reactions to determine if a true allergy exists. Therefore, clinicians should make proper allergy documentation and reporting an integral part of their daily practice. Future research should continue to build on the statistical information and recommendations we have proposed in this manuscript and should be followed up to determine if improvements in allergy documentation occurred.

## Data Availability

All data generated or analyzed during this study are included in this published article (and its supplementary information files). No publicly archived data. Data was derived from the Electronic Medical Records from Medstar Union Memorial Hospital System.
